# An Overview on Coinage Metal Nanocluster-Based Luminescent Biosensors via Etching Chemistry

**DOI:** 10.3390/bios12070511

**Published:** 2022-07-11

**Authors:** Hongxin Si, Tong Shu, Xin Du, Lei Su, Xueji Zhang

**Affiliations:** 1 Beijing Key Laboratory for Bioengineering and Sensing Technology, School of Chemistry and Biological Engineering, University of Science and Technology Beijing, Beijing 100083, China; g20208907@xs.ustb.edu.cn (H.S.); duxin@ustb.edu.cn (X.D.); 2 Shenzhen Key Laboratory for Nano-Biosensing Technology, School of Biomedical Engineering, International Health Science Innovation Center, Research Center for Biosensor and Nanotheranostic, Health Science Center, Shenzhen University, Shenzhen 518060, China; sulei@szu.edu.cn; 3 Guangdong Laboratory of Artificial Intelligence and Digital Economy (SZ), Shenzhen 518060, China

**Keywords:** coinage metal nanoclusters, luminescent probe, etching chemistry, biosensors

## Abstract

The findings from the synthetic mechanism of metal nanoclusters yield the etching chemistry based on coinage metal nanoclusters. The utilization of such chemistry as a tool that can alter the optical properties of metal nanoclusters has inspired the development of a series of emerging luminescent biosensors. Compared with other sensors, the luminescent biosensors have the advantages of being more sensitive, saving time and saving cost. We reviewed topics on the luminescent sensors based on the etching of emissive coinage metal nanoclusters. The molecules possessing varied etching ability towards metal nanoclusters were categorized with discussions of corresponding etching mechanisms. The understanding of etching mechanisms favored the discussions of how to use etching methods to detecting biochemical molecules. The emerging luminescent biosensors via etching chemistry also provided challenges and new opportunities for analytical chemistry and sensors.

## 1. Introduction

Coinage metal nanoclusters (NCs) are typical core-shell structures, which consist of several or even hundreds of metal atoms or ions as the core part, where organic ligand molecules are bonded and act as outer protective groups [1]. The size of metal NCs is generally less than 3 nm, which is close to the electron Fermi wavelength [1,2,3]. Metal NCs therefore exhibit some unique physicochemical properties different from other nanomaterials, such as luminescence, chirality, magnetic, and catalytic properties [4,5,6,7]. Among them, the luminescence of metal NCs is not only adjustable, but also has the characteristics of large Stokes shift, long luminescence lifetime, and good stability [8,9]. In recent years, we have paid more and more attention to its application as an optical material [10,11,12], not only because metal NCs have special optical properties such as strong photoluminescence [8,13,14] and molecular-like absorption [15,16,17,18], but also because metal NCs can achieve atomic precision [4,19,20,21,22]. On the other hand, the ligands of metal NCs are abundant and have significant impact on the structures and properties of the metal NCs. Small molecule ligands such as thiols can freely interact with Au atoms and final are used to yield metal NCs of thermodynamic stability [23]. Macromolecular ligands possess relatively rigid scaffold structures and can offer abundant intramolecular binding sites and confined space for the growth of metal NCs [24]. Therefore, the physicochemical properties of metal NCs are closely associated with metal core and ligand shells [25,26,27].

In the past two decades, highly luminescent metal NCs have been developed as luminescent probes for sensors construction due to their stable luminescence properties and biocompatibility [27,28]. The luminescent biosensor includes two important components: a recognition element and a transducer component [27]. The recognition element can have a specific reaction with the analyte, which determines the selective detection of the analyte by the sensor, while the transducer component is used to convert the chemical signal into a physical signal of luminescence change. Typical luminescent metal NCs are composed of ligand shells and metal cores, both of which can be used as recognition elements. The analyte can undergo metalophilic interactions with metal nuclei, deposit on the metal, or specific reactions that dissolve the metal nuclei [29]. Analytes are also capable of specific reactions with ligand shells through enzymatic reactions, cluster aggregation, and the like [30,31]. Then, a signal of luminescence change is emitted through the transducer component to constitute a completed sensor [27]. The constructed sensors can be used to detect cations (such as Cr^2+^, Cu^2+^, and Hg^2+^) [30,32,33,34,35,36], anions (such as CN^−^ and S^2−^) [37,38,39,40,41] and biomolecules (such as thiols and glucose) [42,43,44,45,46], etc.

Etching technology has a long history and was mostly used for decoration in ancient times [47]. In recent years, metal etching has developed rapidly and is widely used in nanomaterials [48,49,50,51]. We have witnessed the booming development of etching technology as an important factor in the synthesis, preservation, and application of metal nanomaterials [52,53]. The etching process can adjust the shape and size of metal nanomaterials during the synthetic reaction [54,55,56] and has been recognized as an extremely important route in the size-focusing process [4,20]. On the contrary, the storage and application stability of metal nanomaterials are concentrated on the capability to resist etching [57]. In the area of sensor fabrication, etching technology has also been utilized and pushed forward to the rapid development of luminescence biosensors based on metal NCs [1,44]. However, to the best of our knowledge, the topics that focus on the utilization of etching technology in metal NC-based sensor fabrication are still blank.

**Figure 1:**
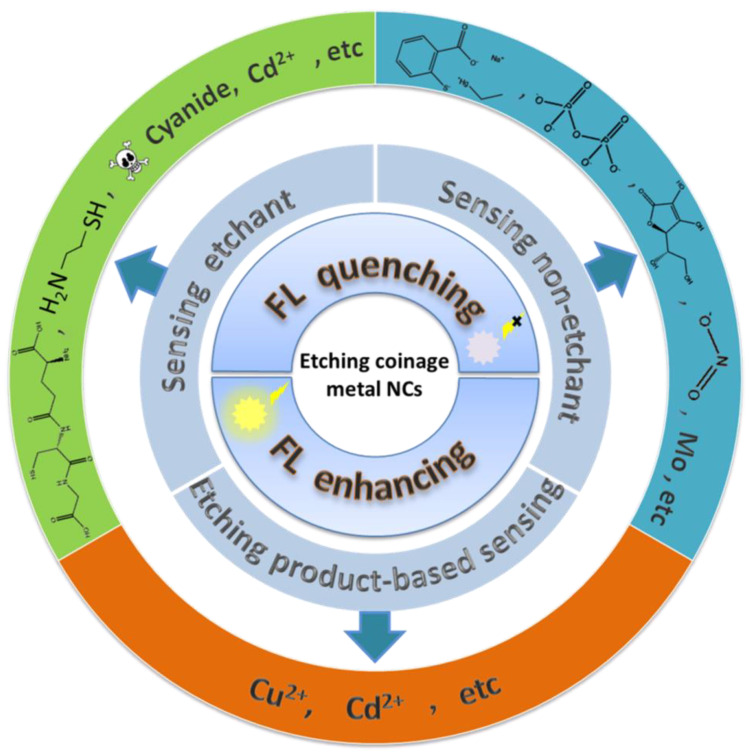
Mechanism diagram of sensor construction based on etched coinage metal NCs.

In this review, the luminescent sensors based on the etching of luminescent metal NCs are systematically investigated and the review topics are spreading with the logical structure as shown in Figure 1. Firstly, the molecules that can etch metal NCs are classified according to the etching functional groups, and related etching mechanisms are discussed. Next, we investigate a wide variety of luminescence biosensors of metal NCs and discuss how to use etching methods to detecting biochemical molecules. Finally, current opportunities and challenges of coinage metal NC-related etching technology spurs the prospect and the new development of valuable biosensors.

## 2. Etching Chemistry of Metal NCs

The strong binding affinity of ligands towards metal NCs is a double-edged sword that can protect metal NCs as well as etch the core metal part of metal NCs. The structure destruction of metal NCs correspondingly affects their physicochemical properties [37,39,42]. These molecules are also called etchants include thiols, cyanide, phosphine compounds, iodine compounds, and some heavy metal ions. The etching chemistry not only establishes the basis for the digestive ripening transformations of metal NCs [58], but also synthesizes new coordination compounds from the debris of etching sculpture [59,60,61].

### 2.1. Thiol-Induced Etching

Thiol functional groups are important components in many biochemical formulations [62] and can also act as building blocks in many chemical reactions [63,64]. Among them, the chemical etching reaction of thiol-induced metal nanomaterials has attracted the attention of researchers due to its wide range of applications, especially the etching of gold NCs (Au NCs) [65,66,67,68,69,70,71,72]. Although etching with excess thiol is widely used, the mechanism is not particularly clear so far [48,73,74,75,76]. Several research groups have attempted to explain the active etching process and necessary conditions of thiol in the reaction. The current consensus is that thiol etchants dissolve metal nuclei and release metal–thiolate complexes.

Early researchers found that thiol-induced etching resulted in a decrease in the core size of the metal NCs and an increase in the amount of metal–thiolate complexes formed during the etching process. Schaaff et al. [77] proposed that the thiol-induced clusters etching process was similar to the formation of Au surface self-assembled monolayers (SAMs). This was based on the observation by WÖll et al. [78] using scanning tunneling microscopy that SAMs were formed by removing gold atoms from the outermost surface of Au. Using techniques such as X-ray diffraction and mass spectrometry, Schaaff et al. [77] directly showed that etching in pure thiol solution could reduce the core size of clusters, and proposed that the mass of clusters decreased while that of Au(I)-SR polymers increased was a plausible mechanism to explain the etching phenomenon. Therefore, rational control of etching conditions may be able to obtain metal NCs with controlled core size. Tsukuda et al. [79] pointed out that etching technology could be used to synthesize thiol-stabilized Au NCs with a controlled core size (Au_n_:SR, _n_ is the core size), and specifically that excess thiols could etch and reduce the core size of Au:SR clusters. Taking the glutathionate (GSH)-stabilized Au NCs (Au_n_(SG)_m_) as an example, it was pointed out that when _n_ < 25, the Au NCs would be etched into smaller clusters by the etchant. When _n_ ≥ 25, the etching products tended to form thermodynamically stable Au_25_:SG clusters.

**Figure 2:**
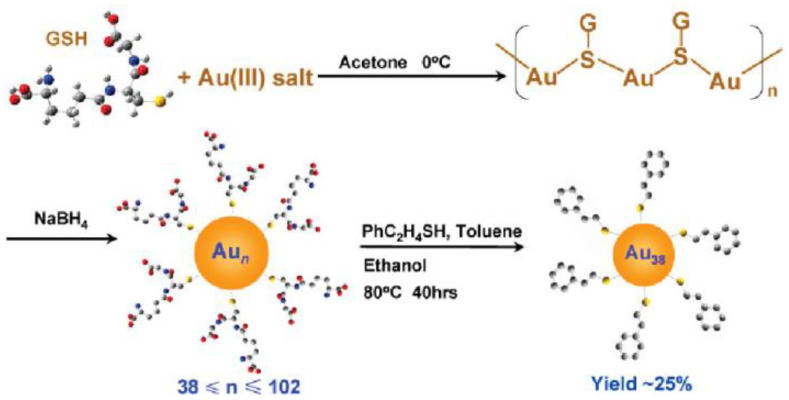
Mechanism diagram of preparation of monodisperse Au_38_(SC_2_H_4_Ph)_24_ clusters by thermal etching of PhC_2_H_4_SH. Reprinted with permission from Ref. [73]. Copyright © 2009 American Chemical Society. Using metal NCs as precursors can also generate sub-nanometer clusters through a thiol-induced interface etching route. Pradeep et al. [66] developed an interface route based on thiol-induced core etching technology, which enabled stable Au_25_ clusters to be etched into sub-nanometer clusters. This was the first report on the preparation of gold sub-NCs by interface etching, and the first report on the synthesis of gold sub-NCs using Au_25_ as a precursor. This report proposed two different etching routes, interfacial etching and single-phase etching, which could generate three different Au NCs, as shown in Figure 3. The team also synthesized two luminescent silver NCs (Ag NCs) using thiol etching technology via the interfacial route [86].

Excess thiol-induced etching transforms metal NCs into a series of atomically precise metal NCs of uniform and stable size, known as “size focusing” [38,48,73,80,81,82,83,84]. Under the action of thiols, pure Au_8_, Au_25_, Au_38_, and other clusters have been successfully synthesized by size-focusing technology. Among them, Tsukuda et al. [79] successfully obtained GSH stable Au_25_ clusters, and pointed out that it was the most thermodynamically stable. Dass et al. [80] obtained Au_36_ clusters by etching a mixture dominated by Au_68_ and Au_102_ clusters using thiophenol. We know that thermal etching induced by excess thiol can selectively etch unstable clusters, resulting in narrow size distribution and thermodynamically stable clusters, but often an Au_38_(SR)_24_ and Au_40_(SR) _24_ mixture. In this regard, Dass et al. [85] proposed a route to obtain Au_38_ or Au_40_ in the etched product on the premise of the etching of single-sized clusters. Jin et al. [73] thermally etched polydisperse Au_n_ clusters (_n_ ≥ 38) with excess phenylethylthiol at 80 °C; that is to say, the size focusing obtained high-purity Au_38_(SC_2_H_4_Ph)_24_ clusters (Figure 2). Since then, Qian et al. [83] also successfully synthesized an Au_38_(SR)_24_ nanocluster using this method and explored its structure and properties. In addition, Jin et al. [82] also found that, when dodecanethiols exchanged ligands with GSH-protected AuNCs, dodecanethiols would etch the gold core, causing secondary growth of the clusters to obtain Au_38_(SC_12_H_25_)_24_ with good dispersion and high purity. Despite the good stability of Au_38_ clusters, Jin et al. [81] found that reacting Au_38_(SCH_2_CH_2_Ph)_24_ with HSPh-tBu at 80 °C for more than 12 h transformed them into new Au_36_ NCs. Dass et al. [84] also obtained Au_99_ clusters by etching Au_144_ clusters with benzenethiol at 80 °C for 3 h.

**Figure 4:**
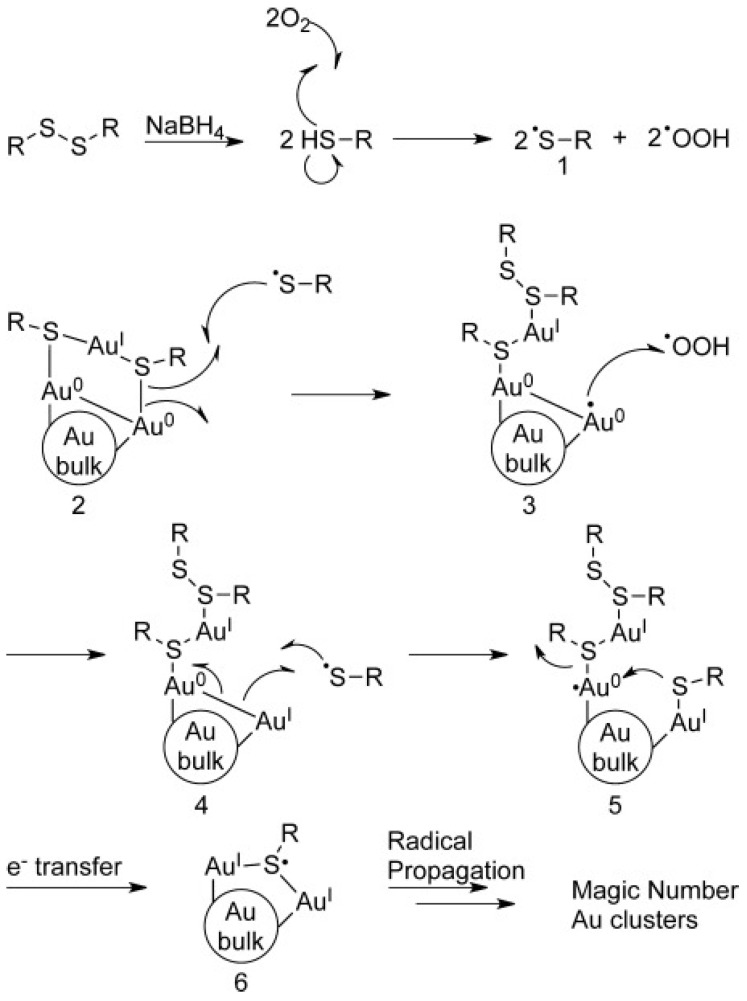
Etching mechanisms dependent on oxygen radicals. Reprinted with permission from Ref. [59]. Copyright © 2015 WILEY-VCH Verlag GmbH & Co. KGaA, Weinheim, Germany.

Thiol etching can be used to prepare sub-nanometer clusters, and then the conditions and mechanism required for thiol etching of metal NCs will be introduced. For the etching of metal NCs induced by thiols, Tsukuda et al. [79] believed that the etching was achieved because thiols could enter the core part of the clusters, and pointed out that oxygen could accelerate the etching process. Chechik et al. [87] pointed out that in the process of ligand exchange between phosphine-based clusters and thiolates, oxygen also played a decisive role. Later, Ackerson et al. [59] also pointed out that the process of etching gold nanoparticles (Au NPs) into smaller Au NCs depended on oxygen, and the free radical character of oxygen played a decisive role (Figure 4)**.** When using thiol as ligand to synthesize Au NCs, the Au (I)-thiolate oligomer was first reduced by reducing agent to form Au NPs, and the disulfide and excess reducing agent generated during this process remained in the solution. Next, the reducing agent would reduce the disulfides to free thiols, and then the oxygen radicals would obtain protons from them to generate thiyl radicals (1) and HOO˙ (previously, several scientific teams confirmed that oxygen could react with thiols to form thiyl radicals [88,89]). Subsequently, the gold–sulfur bond was broken under the action of the thiyl radical, and the core Au^0^ was exposed. The exposed Au^0^ was oxidized to the Au^I^ atom by the previously generated HOO˙ (3). Immediately, the Au^0^-Au^0^ bond was homolytically cleaved to generate (5), and another thiol was now attached to the cluster. The cleavage of Au-S in (5) generated an Au-thiolate monomer and another exposed Au^I^, and then a new Au-S bond was formed (6). So far, the initial cluster was successfully removed from one layer of Au. This cycle could be repeated in the presence of oxygen until all AuNPs were etched to yield thermodynamically stable Au NCs. It can be seen from Figure 4 that the etching cannot proceed without oxygen. Very recently, Xie et al. [90] explained the etching process at the molecular level by monitoring the changes of thiolate-stabilized Au NCs during the etching process in real time by electrospray ionization mass spectrometry.

In addition, some research groups pointed out that the etching phenomenon caused by thiol was affected by pH. Kawasaki et al. [67] found in the preparation of pepsin-mediated Au NCs that if the pH was adjusted from 1 to 9, green-luminescent Au_13_ NCs would generate blue-luminescent Au_5_ and Au_8_ NCs. Subsequently, after exploring the core etching effect of different kinds of alkanethiols on Au_8_, Tseng et al. [91] pointed out that the carboxyl group in the thiol etchant was the key to etch the cluster core, and the core etching effect was enhanced with the decrease of the alkyl chain length in alkanethiols. At the same time, it was pointed out that pH would also affect the etching effect. When the pH was 9 compared to pH 3, the etchant could inject electrons into the clusters more effectively, thereby accelerating the nucleation etching process. Liu et al. [69] also pointed out that the etching effect was pH dependent when using the thiol core etching technique to prepare Ag NCs.

### 2.2. Cyanide-Induced Etching

**Figure 5:**
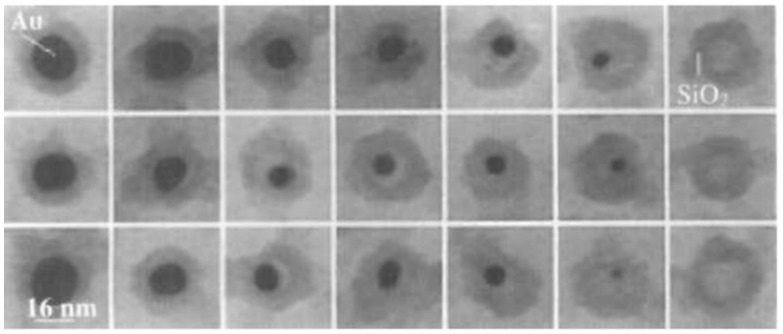
Electron micrographs of three silica-coated gold nanoparticles after adding KCN at 0, 2, 4, 6, 10, and 12 min, respectively. Reprinted with permission from Ref. [93]. Copyright © 1997 Verlag GmbH & Co. KGaA, Weinheim, Germany.

CN^−^ has strong complexing ability with heavy metal ions. In the presence of dissolved oxygen, gold can react with CN^−^ to form Au(CN)_2_^−^, which is the most stable of the Au(I) complexes and has been used for gold extraction for a long time [85]. It is not only able to form Au(CN)_2_^−^, but CN^−^ can also combine with silver, copper, and other metals to form complexes [92]. This specific reaction enables CN^−^ to also have the ability to etch metal nanomaterials. Mulvaney et al. [93] found that CN^−^ could completely oxidize and dissolve the gold core through the etching process when preparing silica-coated Au NPs, as shown in Figure 5. Zeng et al. [94] found that CN^−^ was also able to etch Au@Ag core/shell NPs. Later, Dong et al. [95,96] pointed out that CN^−^ could also etch silver and gold NPs. Likewise, CN^−^ also has the ability to etch metal NCs. For example, Peng et al. [97] found that CN^−^ could be used to etch Ag NCs, and Shamsipur et al. [98] found that CN^−^ could also etch Au NCs. Tian et al. [39] found that CN^−^ was able to etch bimetallic gold–silver NCs and proposed an etching mechanism. The reaction formula after the introduction of CN^−^ into this cluster is as follows:

4Au + 8CN^−^ + O_2_ + 2H_2_O = 4[Au(CN)_2_]^−^ + 4OH^−^ (1)

4Ag + 8CN^−^ + O_2_ + 2H_2_O = 4[Ag(CN)_2_]^−^ + 4OH^−^ (2)

This also shows that CN^−^ can form an Au(CN)_2_^−^ and Ag(CN)_2_^−^ complex through coordination with Au^+^ and Ag^+^ generated by oxygen oxidation in the cluster, so as to realize the etching. During the CN^−^ etching of clusters, not only the presence of oxygen is crucial, but the pH of the solution also affects the etching effect. This is because hydrocyanic acid (HCN) is a weak acid (CN^−^+H_2_O ⇌ HCN+ OH^−^, pKa = 9:36), and when the pH is greater than 9.36, the CN^−^ form is in the majority, which is favorable for etching. Lu et al. [37] used CN^−^ to etch bovine serum albumin (BSA) -protected Au NCs, Chang et al. [98,99] used CN^−^ to etch copper clusters (Cu NCs), and other research teams [98] also hold the same view.

**Figure 6:**
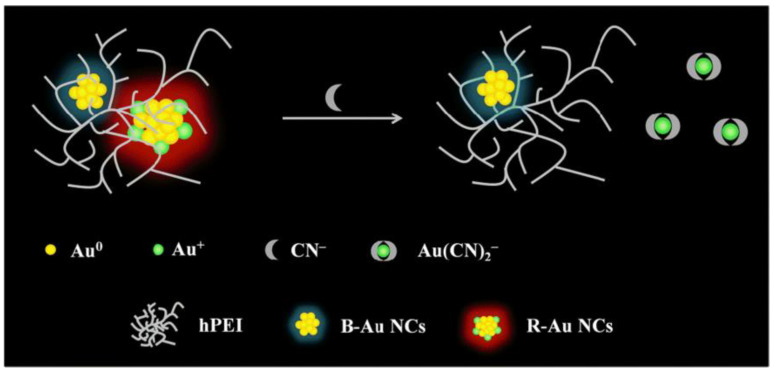
Schematic diagram of constructing ratiometric CN^−^ sensing with CN^−^ etching DE-AuNCs. Reprinted with permission from Ref. [40]. Copyright © 2020, Springer-Verlag GmbH Germany, part of Springer Nature.

Some researchers have proposed that the surface valence state of the clusters can be used to explain the role of CN^−^ in the induced etching of metal NCs. To this end, Yuan et al. [40] prepared dual-emissive gold NCs (DE-AuNCs), which had different emission characters and surface valence states, and attempted to illustrate the importance of surface valence states for driving CN^−^ etching. Among them, R-Au NCs had a high content of surface Au(I) with red emission, while B-Au NCs had no Au(I) surface but blue emission (Figure 6). After the introduction of CN^−^, CN^−^ was able to complex with Au(I) on the surface of R-Au NCs, that is, etching occurred. Since the surface of B-Au NCs did not have Au(I), etching could not be carried out. The change of luminescence before and after etching confirmed this conjecture.

### 2.3. Phosphine Compound-Induced Etching

**Figure 7:**
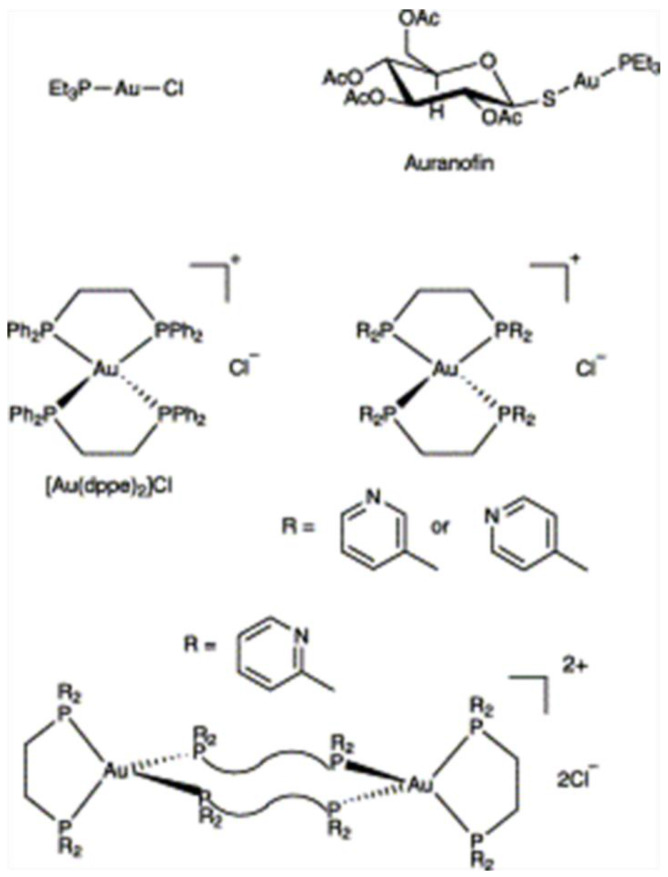
Chemical structures of some gold(I)–phosphine complexes. Reprinted with permission from Ref. [101]. Copyright © 1981 American Chemical Society.

Phosphine compounds can coordinate with a variety of metals and have wide application prospects. For example, diphosphine can coordinate with Cu(I), and the resulting complexes have fascinating phosphorescence properties based on the MLCT character [100]. Phosphine compounds can also coordinate with gold, such as alkynyl phosphine, and tertiary phosphines. Figure 7 shows the molecular structures of some gold(I)–phosphine complexes [101]. Ott et al. [102] also proposed that alkynyl (triphenylphosphine) could coordinate with gold. It was also pointed out that tertiary phosphines were the most widely studied because they were more stable [103]. Tertiary phosphines, like other metal ligands (such as thiols), can be used as ligands for the synthesis of metal NCs or metal NPs. At present, several teams have synthesized atomically precise Au NCs using phosphine compounds as ligands [104,105,106,107,108,109,110]. However, Lotnyk et al. [111] reported that water-soluble tertiary phosphines could reduce Au(III) to Au(0) and promote the oxidative dissolution of gold(0) nanocrystals by forming phosphine–Au(I) complexes. At the same time, studies have shown that some phosphines, such as triphenylphosphine (PPh_3_), can not only be used as protective agents during the synthesis of stable NCs, but also can etch the metal core part of the clusters [112,113,114,115].

**Figure 8:**
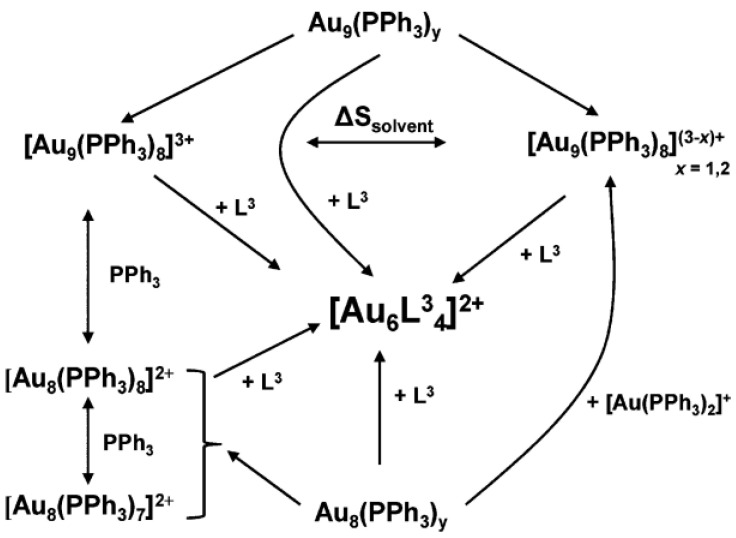
Conversion of Au NCs synthesized with PPh_3_ as ligand under phosphine etchants. Reprinted with permission from Ref. [112]. Copyright © 2011 American Chemical Society.

Etchants such as phosphine compounds enable cluster-to-cluster interconversion. Bour et al. [115] pointed out that Au NCs compounds could undergo cluster-to-cluster interconversion at a fast rate; that is, the initial cluster was partially or completely broken and then reassembled into a new cluster. Phosphine compounds as etching agents can achieve this conversion. Au_9_ NCs synthesized with PPh_3_ as ligands were etched by excess PPh_3_ and converted into Au_8_ NCs, and then etched by Ph_2_PCH_2_PPh_2__,_ where they were converted into Au_5_ NCs. The Au_9_ NCs are also etched by 1,3-bis(diphenylphosphino)propane (L^3^) and converted into Au_6_ NCs, as shown in the Figure 8 [112]. Later, Konishi et al. [114] pointed out that Au_6_ NCs could also be etched by a diphosphine ligand, which could recombine and generate two new Au_8_ NCs to complete the conversion between clusters. In the same year, Hudgens et al. [112] clearly proposed for the first time that PPh_3_ could be used not only as a protective ligand, but also as an active etch agent for the size selective synthesis of small clusters, and provided evidence through colorimetry. Following this, Hudgens et al. [113] also emphasized the essential importance of etching in the size-selective synthesis of monodisperse L^3^-protected gold clusters. Our team found that tris(2-carboxyethyl)phosphine (TCEP) also had etching properties and could etch BSA-Au NCs [61,116]. Through the UV-vis adsorption spectrum, X-ray photoelectron spectroscopy (XPS), and other technologies, it has been proven that TCEP can etch the gold (0) core in BSA-Au NCs, dissolve the Au NCs, and generate Au(I) complexes.

### 2.4. Iodine Compound-Induced Etching

**Figure 9:**
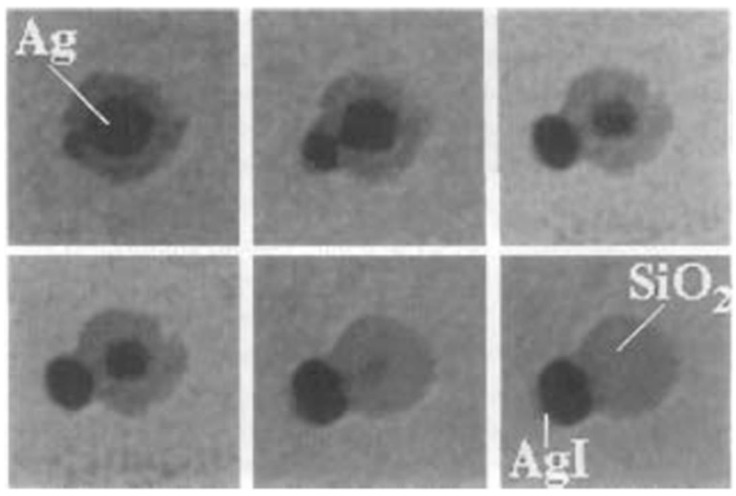
Electron microscope images of silica-coated silver nanoparticles exposed to I_2_ for different times. Reproduced with permission from [93]. Copyright © 1997 Verlag GmbH & Co. KGaA, WeinheimNumerous studies have shown that I_2_ has the ability to etch coinage noble metal NCs. Mo et al. [119] proposed that I_2_ has an oxidative etching effect on lysozyme(Lys)-protected silver NCs (Lys-AgNCs), and I_2_ was obtained through the redox reaction of iodate (IO_3_^−^) and ascorbic acid (AA). The reaction equations are as follows.

It has been reported that iodine also has the ability to etch metal NCs [117,118,119]. Mulvaney et al. [93] found that molecular iodine(I_2_) could completely oxidize the silver core through the etching process when preparing silica-coated Ag NPs. The I_2_ could react with the silver nucleus in the silver nanoparticles to generate AgI, which gradually made the silver nucleus smaller and eventually disappeared completely. This process was observed by transmission electron microscopy (TEM) (Figure 9). Subsequently, Wang et al. [120] also confirmed that I_2_ could chemisorb with Au NPs, and observed fusion/fragmentation and aggregation of Au NPs by TEM. In addition, Chen’s team found that I_2_ also had an etching effect on gold nanorods [121,122]. Additionally, Huang et al. [123] used techniques, such as scattered-light dark-field microscopic imaging (iDFM), to monitor the reaction process in real time, revealing the etching process of I_2_ on NPs of various shapes. The phenomenon that iodine can etch metal NPs has aroused the strong interest of scientific researchers. If I_2_ can cause the same etching phenomenon of metal NCs, then a variety of luminescence detection methods can be developed due to the luminescent properties of the clusters. Therefore, researchers have turned their attention to whether I_2_ can etch metal NCs. Subsequently, multiple research teams showed that I_2_ could etch metal NCs.

2IO_3_^−^ + 5C_6_H_8_O_6_ + 2H^+^ →5C_6_H_6_O_6_ + I_2_ + 6H_2_0 (3)

2Ag + I_2_ → 2AgI↓ (4)

I_2_ is also capable of etching Au NCs, which Yan’s team conducted a lot of research on [117,118,124]. They pointed out that iodide(I^−^) was oxidized to I_2_ by S_2_O_8_^2−^ under the catalysis of Cu^2+^, and the generated I_2_ could etch the BSA-Au NCs [124]. I^−^ itself did not etch the clusters, but the combination of I_2_ and I^−^ formed by oxidation was a strong etchant for Au(0) according to the following reaction:

I₂ + I^−^ → I_3_^−^ (5)

I_3_^−^+ I^−^ + 2Au(s) → 2AuI_2_^−^ (6)

AuI_2_^−^ + I_3_^−^ → AuI_4_^−^ + I^−^ (7)

Later, the team also pointed out that the I₂ obtained by catalyzing the I^−^ into I₂ process by nitrite and molybdenum (Mo) could also etch Au NCs [117,118].

Not only does I_2_ have the ability to etch metal NCs, but Yan et al. [125] found that iodate (IO_3_^−^) can also oxidize and etch BSA-AuNCs to quench their luminescence. Through dialysis, it was determined that IO_3_^−^ could directly etch the gold core of the clusters. In addition, the strong oxidizing ability of IO_3_^−^ was enhanced under acidic conditions, which could improve the etching effect. The research group also pointed out that extra I^−^ could enhance the etching process of IO_3_^−^ to Au NCs. Because I^−^ would be oxidized by IO_3_^−^ to I_2_ (**1**), I_2_ and I^−^ generated more soluble triiodide ion(I_3_^−^) (**2**), the combination of I_3_^−^ and I^−^ not only strongly etched Au(0) (**3**), but also oxidized Au(I) to soluble tetraiodoaurate (III) complexes (**4**), and the reaction equations are as follows:

5I^−^+ IO_3_^−^ + 6H^+^ → 3I_2(s)_ + 3H_2_O (8)

I_2(s)_ + I^−^ → I_3_^−^ (9)

2Au_s_ +I^−^ + I_3_^−^ → 2AuI_2_^−^ (10)

Au(I) + I^−^ + I_3_^−^ → AuI_4_^−^ (11)

2AuI_2_^−^+ 2I_3_^−^ → 2AuI_4_^−^ + 2I^−^ (12)

### 2.5. Heavy Metal Ion-Induced Etching

Some heavy metal ions, such as chromium ions (Cr(VI)), one of the two most common valence states of Chromium in nature, have also been found to have the ability to etch metal nanomaterials [126,127]. Usually, Cr(VI) could play a strong oxidative effect to etch metal nanomaterials in the presence of bromide ions (Br^−^) [32,128]. Li et al. [129] pointed out that Cr(VI) could selectively etch gold nanorods with lower aspect ratio, and pointed out that the decrease of pH value, the increase of temperature, and the presence of Cl^−^ or Br^−^ could promote the etching process. Xin et al. [130] and Li et al. [131] found that, in the presence of Br^−^, Cr(VI) could also oxidize and etch bimetallic core-shell metal NPs. Of course, Cr(VI) could also etch NPs such as gold and silver with the help of Br^−^ [126,127,128,132].

After that, researchers focused on the etching of coinage metal NCs by Cr(VI). Guo et al. [32] pointed out that Cr(VI) could etch BSA-AuNCs. Normally, Cr(VI)/Cr(III) has a lower electron potential than Au(I)/Au(0), so Cr(VI) could not etch gold [128,129,130]. After adding Br^−^, Br^−^ would form AuBr_2_^−^ compounds with gold and reduce the electron potential of Au(I)/Au(0), so that Cr(VI) could etch Au(0) in BSA-Au NCs. Zhang et al. [133] pointed out that Cr(VI) could etch polyethyleneimine-protected Ag NCs, and the etching could also be accomplished by oxidizing Cr(III) to Cr(VI) using H_2_O_2_ under alkaline conditions.

## 3. Sensor Construction Based on Metal Nanocluster Etching

The etchants can modify or even destroy the structure of metal NCs and therefore cause the luminescent properties of metal NCs to be changed or quenched. The luminescent signal alteration can be used to construct optical biosensors. The metal NCs act as recognition components and recognize the analyte through a specific reaction, and then convert the chemical signal into a physical signal of luminescence change, which is emitted by a transducer component. Luminescence quenching and luminescence enhancement are two common strategies for constructing biosensors based on metal NCs etching and can quantify the analyte through the changing intensity of luminescence.

### 3.1. Etchant Detection

Etched metal NCs can be used to construct luminescent sensors for the detection of etchants. Firstly, the biosensors for detecting thiols are introduced. Several studies have shown that GSH can etch Au NCs. Chen et al. [42] pointed out that GSH could etch the core of Au8 clusters synthesized with (Lys VI) as a ligand and make them quench their luminescence. In other words, the invisible etch reaction triggered by GSH was converted into luminescence signals that could be recognized by naked eyes, and a luminescence sensor for GSH detection could be constructed based on this. The luminescence signal can be used as a basis for constructing a luminescence biosensor to detect GSH. Wu et al. [44] constructed a sensor to detect lactate dehydrogenase (LDH). LDH can be used as a biomarker for common diseases, and its surface contains free thiol groups and thus also has the ability to etch metal NCs. This report took this as an entry point, using free thiols in LDH to etch Au NCs protected by adenosine monophosphate (AMP), and then the luminescence emission of AMP-AuNCs could be quenched. This detection method using etching metal NCs to construct a luminescence sensor has higher sensitivity and lower detection line, which is in line with the scope of clinical diagnosis.

**Figure 10:**
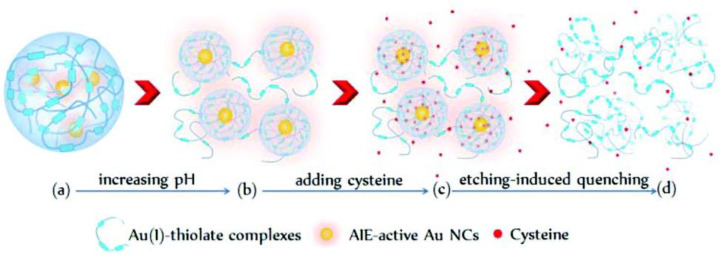
Schematic illustration of cysteine-induced etching and luminescence quenching of AIE-Au NCs. (**a**) the AIE-active Au NCs encapsulated by the Au(I)-thiolate complex network; (**a**→**b**) Increasing the pH prompted the disassembly of the Au(I)–thiolate complex network; (**b**→**c**) alkaline pH allowed cysteine to enter the Au NCs; (**c**→**d**) cysteine molecules etched the Au(0) cores. Reprinted with permission from Ref. [46]. Copyright © 2019 RSC Pub.

In addition, our group found that cysteamine could etch BSA-stabilized Au_25_ NCs to quench their luminescence, while cysteine and even other 19 natural amino acids did not have this function [45]. A luminescent biosensor capable of selectively detecting cysteamine was developed. Later, we [46] found that cysteine could etch Au NCs with aggregation-induced emission (AIE) property to quench their luminescence, as shown in Figure 10. The AIE-type Au NCs that we synthesized contained a large amount of Au(I)–thiolate complexes. Since the permeability of Au(I)–thiolate complexes to cysteine was affected by pH, pH would also affect the etching effect of cysteine on the Au NCs. The experimental results showed that low concentrations of cysteine could enhance the luminescence of the Au NCs, but high concentrations of cysteine could still quench the luminescence, and the higher the pH of the solution, the more obvious the etching effect. Therefore, we constructed a cysteine biosensor based on the etching of AIE-Au NCs by cysteine under alkaline conditions. In addition to building sensors to detect thiols, many teams have also built sensors to detect other types of etchants, which are described in turn below.

Cyanide is widely found in nature and has a variety of uses. It is indispensable in industry, mining, and organic synthesis [134,135]. However, cyanide is highly toxic: CN^−^ has super complex ability to heavy metal ions, can combine with intracellular metal ions, or destroy the human respiratory chain and other physiological functions by inhibiting enzyme activity, endangering life. In human activities such as gold mining, electroplating, etc., cyanide leakage can cause serious harm to water and soil [136]. At present, researchers have taken various measures to reduce the harm caused by cyanide leakage, such as the use of biodegradation [137] and biosorbents [138]. At the same time, how to detect cyanide quickly and effectively is also very important. Due to the strong complexing ability of CN^−^ with heavy metal ions, this specific reaction can etch metal NCs, so it can be used to design CN^−^-sensing probes with metal nanomaterials.

Etching metal NCs with cyanide can be used to construct biosensors to detect CN^−^. This photochemical sensing method stands out among numerous approaches such as potentiometric, amperometric, and titrimetric approaches due to its simplicity, sensitivity, and efficiency. It has been introduced that cyanide can induce the etching of metal NCs, so the purpose of detecting cyanide can also be achieved by using the luminescence quenching of metal NCs after etching as a signal. Lu et al. [37] successfully constructed a nano-luminescent sensor for detecting cyanide using etching technology, which can be used for the detection of cyanide in water samples, food, soil, and other samples. Subsequently, Dong et al. [38] found that under the etching effect of cyanide, Lys-Au NCs would quench the red luminescence of the Au NCs itself, and appeared as blue luminescence, and the luminescence intensity was linearly related to the concentration of CN^−^. Thus, the sensor constructed could be used to detect cyanide in the environment. Li et al. [39] prepared bimetallic gold–silver NCs with stronger luminescence, which were highly sensitive and selective to cyanide. Sensors built on this basis can be used not only for sensing cyanide, but also for temperature sensing and cell imaging. Yuan et al. [40] also constructed a novel ratiometric sensor to detect CN^−^ based on the fact that dual-emissive Au NCs could be etched by CN^−^. This sensor saved time and was more sensitive than previous chemical sensors that detected CN^−^ [139,140,141]. In addition, the preparation of sensors based on cyanide etched clusters was also reported by other groups [97,98,99,142,143].

Cr(III) in the correct amount is an essential nutrient; however, when present in excessive concentrations, it will damage the cell structure and cause harm to the human body [144]. In contrast, Cr(VI) is more dangerous because of its strong oxidizing properties, and once it enters the human body, it will cause damage to organs such as the liver [145,146]. Using large-scale instruments, such as X-ray luminescence [147] and atomic absorption spectroscopy [148], can effectively detect Chromium, but the cost is high and cumbersome, and it is not suitable for real-time monitoring. Using the etching ability of Cr(VI) to metal NCs to develop a sensitive luminescence method to detect Cr(VI) is efficient, simple, and more practical.

**Figure 11:**
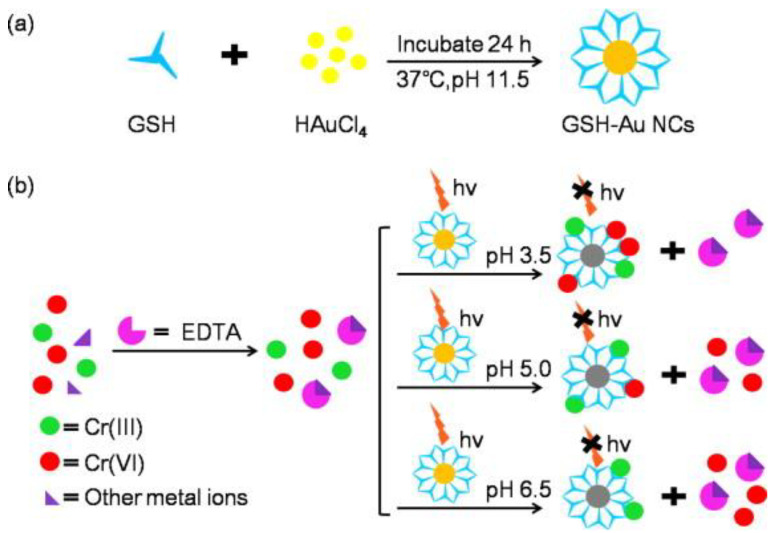
Schematic diagram of synthesis of GSH-Au NCs (**a**) and realization of detection of Cr(III) and Cr(VI) (**b**). Reprinted with permission from Ref. [33]. Copyright © 2013 Elsevier B.V.

**Figure 12:**
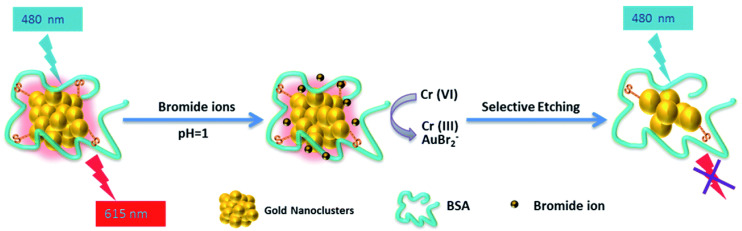
Mechanism of Cr(VI) selectively etching BSA-Au NCs and quenching their luminescence. Reprinted with permission from Ref. [32], Copyright © 2016 The Royal Society of Chemistry.

**Figure 13:**
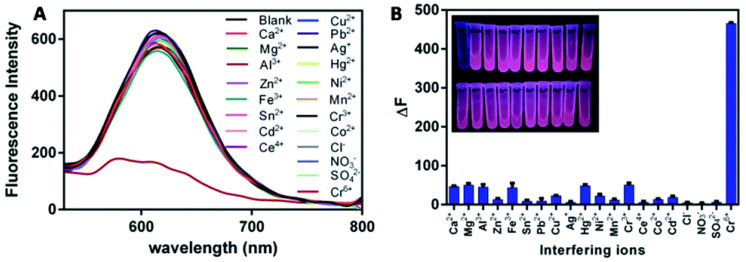
Specific determination of Cr(VI)-etched BSA-Au NCs. (**A**) Luminescence emission spectra of BSA-Au NCs after adding different ions. (**B**) The degree of luminescence change of BSA-Au NCs after adding different ions (the inset is the imaging under UV light). Reprinted with permission from Ref. [32]. Copyright © 2016 The Royal Society of Chemistry.

Next, we introduce the construction of a sensor for chromium ion detection based on etching metal NCs. Liu et al. [33] constructed a nano-luminescence sensor for the detection of Cr (III) and Cr(VI) in water samples. The schematic diagram is shown in Figure 11. In this experiment, luminescent GSH-Au NCs were first synthesized, and then it was found that the etching ability of Cr(III) and Cr(VI) on the Au NCs was pH dependent. At pH 6.5, since Cr(VI) did not have the ability to etch the Au NCs, Cr(III) could be directly detected. At pH 3.5 and 5.0, there was almost no difference in the etching ability of Cr(III), Cr(VI) could be detected according to the change of relative luminescence intensity, and the possible interference caused by other metal ions was eliminated by adding Ethylenediaminetetraacetic acid (EDTA) during the etching process. Guo et al. [32] designed a luminescence method to detect Cr(VI) and introduced the etching mechanism. Namely, selective etching of BSA-Au NCs using the strong oxidative power of Cr(VI) in the presence of Br^−^, which quenched the luminescence of BSA-Au NCs themselves, and to achieve the purpose of detecting Cr(VI). First, the BSA-Au NCs would emit red light at 615 nm when excited at 480 nm. Normally, Cr(VI)/Cr(III) had a lower electron potential than Au(I)/Au(0), so Cr(VI) could not etch gold [126,129,130]. After Br^−^ was added, Br^−^ would form AuBr_2_^−^ compounds with gold to reduce the electronic potential of Au(I)/Au(0), so that Cr(VI) could etch the gold in BSA-Au NCs, resulting in the change of the structure of the gold cluster and the luminescence quenching (Figure 12). The content of Cr(VI) in the sample could be calculated from the change in the luminescence intensity of BSA-Au NCs. Specificity was also tested in this work, as shown in Figure 13. At present, this method can be applied to monitor Cr(VI) in river water, and its feasibility has been confirmed.

Biosensors constructed by etching metal NCs are not always signaled by the appearance or quenching of luminescence after etching of metal NCs. Wang et al. [149] pointed out that certain thiols could enhance the photoluminescence (PL) of Au NCs synthesized with cytidine as a ligand, such as GSH. We generally believe that the PL enhancement is due to the aggregation-induced luminescence of the NCs, that is, the high content of metal(I)–thiolates complexes or complex aggregates on the surface of the NCs [150,151]. Through a series of experiments, Wang et al. [152] confirmed that this finding was not true, but that the etching of the Au NCs by GSH resulted in the formation of smaller and more luminescence Au species. Because of this, the content of GSH could be determined according to the maximum luminescence increase rate, and it could also be extended to identify the activity of glutathione reductase, which has bright application prospects in clinical and other medical fields. A comparison of luminescence sensors based on the etching of coinage metal nanoclusters and other detection methods is shown in Table 1.

### 3.2. Indirect Sensing Method Using Etchant

**Figure 14:**
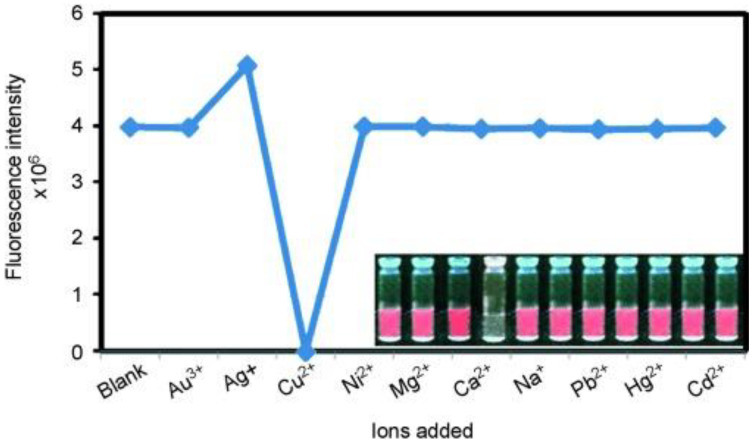
Changes in the luminescence intensity of Au_23_ NCs after introducing different cations (the inset is the picture under UV light). Reproduced with permission from [66]. Copyright © 2009 WILEY-VCH Verlag GmbH & Co. KGaA, Weinheim, Germany.

Using the properties of the etchant, sensors can be constructed to detect the etchant, which has been described in detail before, but is by no means limited to this. For example, Pradeep et al. [66] pointed out that Au_23_ NCs generated by etching Au_25_ NCs with thiols could be used to construct biosensors for sensing Cu^2+^ ions. This is because the Au_23_ NCs produced after etching had luminescent properties and were soluble in water, and the addition of Cu^2+^ could quench their luminescence, as shown in Figure 14. Thus, the constructed biosensor could specifically recognize Cu^2+^ and could be extended to the detection of water samples. Tseng et al. [91] constructed a biosensor for thimerosal detection based on thiosalicylic acid (TSA)-etched Lys VI-protected Au_8_ NCs. Thimerosal contains TSA and the ethylmercuric (EtHg) ion, which can be used as antibacterial preservatives and are often used in vaccines [161], but vaccines containing thimerosal can affect neurodevelopment. The biosensor constructed by Tseng et al. could sensitively and rapidly analyze the content of thimerosal in the drug by using the luminescence change of the clusters as the signal. Lin et al. [71] proposed that the etching-based process could also be used to fabricate a novel biosensor for the detection of biomolecules, such as alkaline phosphatase (ALP) and pyrophosphate (PPi), and introduced Cu^2+^ in the process. Firstly, Au NCs with a blue-green emission were prepared through the nuclear etching process induced by cysteine, and then the luminescence of Au NCs quenched by Cu^2+^ was recovered due to the coordination and binding of PPi to Cu^2+^, thus realizing the detection of PPi. ALP could hydrolyze PPi and release the previously bound Cu^2+^, which re-quenched the luminescence and could identify the activity of ALP. Konishi et al. [114] also prepared a luminescent sensor for sensitive detection of mercury ions using Au_8_ NCs generated by a diphosphine ligand etching.

In the process of I_2_ etching of metal NCs, it is usually necessary to use catalysts to catalyze various iodides to generate I_2_ or to use redox reactions to generate I_2_, so biosensors that detect non-etchants can be constructed. For example, Shao et al. [119] constructed a luminescent biosensor capable of sensitively detecting AA. This was because AA could react with IO_3_^−^ to generate I_2_, which could etch Lys-Ag NCs and make it quench the luminescence to achieve the purpose of detecting AA. Yan et al. [124] developed a biosensor that used BSA-Au NCs as luminescent probes to sense Cu^2+^. Cu^2+^ was used here to catalyze the reaction between potassium persulfate and potassium iodide to generate I_2_. Previous reports confirmed the strong and effective catalytic activity of Cu^2+^ on persulfate [162]. The degree of luminescence quenching of BSA-Au NCs by the generated I_2_ had a linear relationship with the content of Cu^2+^, so the detection of Cu^2+^ could be realized. Yan et al. also pointed out in later work that BSA-Au NCs could also be used as luminescent probes to construct biosensors for the detection of nitrite [117] and Mo [118], in which nitrite quenched the luminescence by catalyzing the reaction of potassium iodide and potassium bromate to generate I_2_, and Mo quenched the luminescence by catalyzing the reaction of H_2_O_2_ and potassium iodide to generate I_2_.

### 3.3. Etching Product-Based Sensors

**Figure 15:**
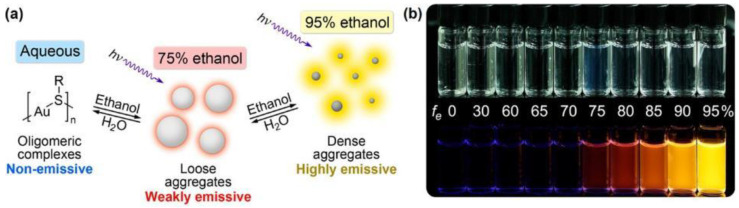
Solvent-induced Au–thiolate complexes aggregation-induced emission (AIE) phenomenon. (**a**) Schematic diagram of solvent-induced AIE properties of Au(I)−thiolate complexes. (**b**) Digital photos of Au(I)−thiolate complexes in different fraction of ethanol under visible (top row) and UV (bottom row) light. Reprinted with permission from Ref. [150]. Copyright © 2012 American Chemical Society.

Taking thiol etching of Au NCs as an example, we already know that non-luminescent Au(I)–thiol complexes will be formed after etching, which has been mentioned in many reports [59,60,61,163,164]. However, we often focus on the changes of the clusters during the etching process, ignoring the Au(I)–thiol complexes released during this process, but they are not without application. The study found that although the Au(I)–thiol complex did not emit light, it could aggregate and emit light after adding a weak polar solvent or divalent cation (such as Cd^2+^) to its aqueous solution; that is, it could produce aggregation-induced emission (AIE) effect. In the early, Xie et al. [150] pointed out that weakly polar solvents (such as ethanol) could induce aggregation of Au–GSH complexes, as shown in Figure 15a, when the volume fraction of ethanol was 75%, the Au–thiolate complexes began to aggregate and emit weak light. When the volume fraction of ethanol continued to increase to 95%, the aggregation became denser and there was strong emission. The two rows of Figure 15b reveal the state of the complex solution under visible light (upper row) and UV light (lower row) with increasing ethanol volume fraction, respectively. When the volume fraction of ethanol was 75% due to the formation of aggregates, the solution was turbid and initially luminescent, and when the volume fraction of ethanol increased to 95%, the solution became clear again and had strong luminescence, so it could be adjusted by adjusting the weak polar solvent in water volume fraction to control the intensity of AIE. Later, we [60] used the property of Cd^2+^ to enable aggregation-induced luminescence of Au(I)–thiolate complexes to prepare a novel colorimetric sensor for the detection of Cu^2+^. First, non-luminescent Au(I)–thiolate complexes were obtained by etching BSA-Au NCs with cysteamine, and then Cd^2+^ was added to aggregate these complexes to produce intra-aggregates with good water solubility and phosphoresce. The phosphorescence emission of intra-aggregates was affected by pH and Cu^2+^, which could be quenched by Cu^2+^. Since there was a linear relationship between the concentration of Cu^2+^ and the degree of phosphorescence quenching, the sensitive detection of Cu^2+^ was realized. Our team also pointed out that Cd^2+^ could also aggregate the Au(I) complexes generated after the etching of BSA-Au NCs by TCEP, stimulating their AIE properties [61]. Effective utilization of the AIE properties of Au(I) complexes generated after etching can not only develop new sensors, but also be used to prepare other luminescent materials.

## 4. Conclusions and Outlook

In this paper, the composition and properties of coinage metal NCs are briefly introduced. The properties of metal NCs largely depend on their structure and size. Studying the crystal structure of metal NCs is undoubtedly beneficial to explain the relationship between their structure and properties. However, it is challenging to obtain the crystal structure of metal NCs with the current technology. In addition, how to obtain metal NCs with ideal properties has also become a critical problem that has to be considered. The present research results show that the structure and size of metal NCs can be controlled by adjusting the reaction process and conditions, so as to obtain ideal metal NCs. Secondly, the etching phenomenon of coinage metal NCs is described in detail. Etching techniques can be used both to generate atomically precise sub-NCs and to nuclear etching coinage metal NCs, destroying the structure of the metal NCs themselves, thereby affecting their properties. As explained in the second part of this paper, thiol etchants have the above two functions, while other etchants such as cyanide are mainly used to nuclear etching coinage metal NCs. However, etching mechanisms, especially thiol-induced etching of emissive or atomically precise metal NCs, are far from clear.

Finally, the sensor based on the etching of coinage metal NCs is introduced, and the research progress of the sensor based on the etching of coinage metal NCs and its application is reviewed. Metal NCs have luminescence properties due to size effect. The etching of coinage metal NCs by etchants such as thiols will destroy the structure of the clusters and quench the luminescence of clusters themselves. Additionally, after etching, smaller sub-NCs are formed and the luminescence is enhanced. The decrease or increase of luminescence signal can be used to construct sensors to detect etching agents and other molecules. The sensors constructed thus not only have great application prospects in chemical detection, bioactive molecule detection, food safety detection, clinical diagnosis, etc., but also provide new ideas for the potential application of metal NCs in biosensing. This is enough to arouse the enthusiasm of researchers.

At present, the sensing technology based on etching luminescent metal NCs has gradually converged into a characteristic research topic. From the point view of coordination chemistry, the etching processes essentially involve the coordinating interaction between etchants and metals. Thus, the expanding of metal NC library is expected to enrich the etching chemistry that can be used for sensor fabrication. The mainstream of future development of sensors is intelligent, which should be accomplished via multidisciplinary cooperation. The introduction of computing science, physics, and electronic engineering will favor the development of instrumented, interconnected, and intelligent sensors based the etching chemistry of metal NCs.

## Figures and Tables

**Figure 3:**
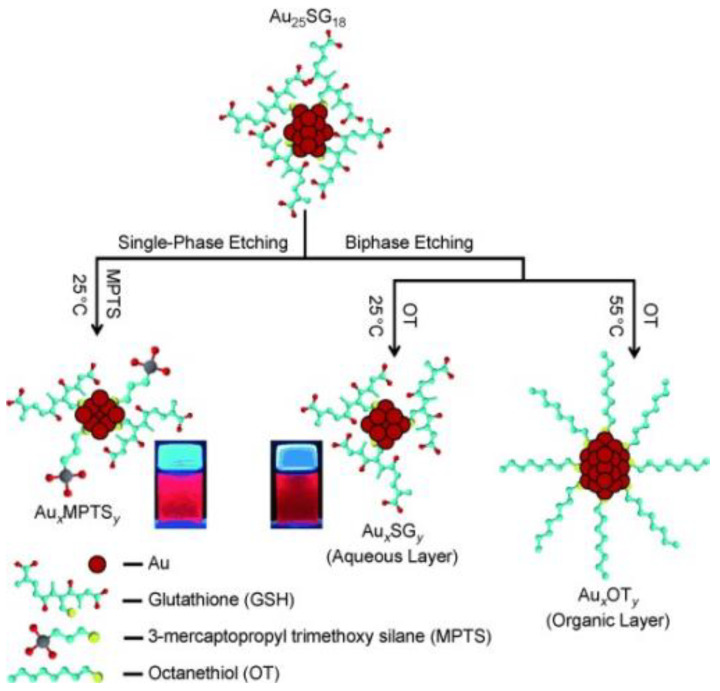
The interface route of thiol etching Au_25_SG_18_. Reprinted with permission from Ref. [66], Copyright © 2009 WILEY-VCH Verlag GmbH & Co. KGaA, Weinheim, Germany.

**Table 1:** Comparison of luminescence sensors based on the etching of coinage metal nanoclusters and other detection methods.

**Target**	**Probe/Instrument**	**Strategy**	**LOD** **(nM)**	**Linear Range (μM)**	**Advantages**	**Disadvantages**	**Reference**
GSH	AuCyt NCs	Fluorescence (GSH etched AuCyt NCs to generate highly fluorescent Au species)	2	0.02–3	Highly sensitive detection, could be extended to detect the glutathione reductase activity	Unable to detect on-site	[149]
	CPR	FRET ^1^ (GSH broke the link between fluorophores, inhibiting FRET)	30	None	Sensitive and selective detection	Probe preparation was complex	[153]
	Lyso-O-NBD	Fluorescence (GSH reacted with Lyso-O-NBD to produce blue fluorescence)	39	0–5	Sensitive and selective detection	Probe preparation was complex	[154]
CN^−^	DE-Au NCs	Ratiometric fluorescence (CN-drove etching DE-Au NCs based on surface valence state)	10	0.02–1	Highly sensitive and selective detection, could be used for river water and urine sample analysis	Unable to detect on-site	[40]
	Molecular Au (I) cluster	Fluorescence (In situ formation of phosphorescent molecular gold(I) cluster)	80	0.16–50	Sensitive detection	Unable to detect on-site	[155]
	β-CD-Au NPs ^2^	Colorimetric (Cyanide etchedβ-CD-Au NPs)	93	4.5–99	Could quickly detect water samples on-site	In contrast, it was not sensitive enough	[156]
	PS 20-Au NP-FITC ^3^	Dual fluorescence–colorimetric assay (cyanide etched PS 20-Au NP-FITC)	100	0–7	Selective detection, cost-effective	Not sensitive enough	[157]
Cr^6+^	BSA-Au NCs	Fluorescence (Cr^6+^ etched BSA-Au NCs)	0.6	0.001–2.5	Highly sensitive detection, simple, short detection time	The quantum yield of BSA-Au NCs was not high	[32]
	X-ray fluorescencespectra	Kβ emission spectra for Cr^6+^ compounds	None	None	Could detect Cr^6+^ compounds	high cost	[147]
	SRBH ^4^	Fluorescence (Reaction between potassium dichromate and non-fluorescent SRBH to produce highly fluorescent Rhodamine B)	1.5	0.01–0.3	Sensitive detection	Only used to detect CrO_4_^2−^	[158]
	CdTe@SiO_2_ and RhB ^5^	FRET (Cr^6+^ and RhB electrostatically attract)	6.2	0.02–0.3	Sensitive detection	Unable to detect on-site	[159]
	AA-capped Ag NPs	Colorimetry (Crosslinking of Cr^6+^ reduction products with AA caused AA-capped Ag NPs to aggregate)	50	0.08–1.84	Sensitive detection, simple	In contrast, it was not sensitive enough	[160]

^1^ fluorescence resonance energy transfer. ^2^ β-Cyclodextrin-protected gold nanoparticle. ^3^ AuNPs decorated with Fluorescein isothiocyanate and polysorbate 20. ^4^ Salicylaldehyde rhodamine B hydrazone. ^5^ Rhodamine B.
